# Changes in air pollution due to COVID-19 lockdowns in 2020: Limited effect on NO_2_, PM_2.5_, and PM_10_ annual means compared to the new WHO Air Quality Guidelines

**DOI:** 10.7189/jogh.12.05043

**Published:** 2022-11-21

**Authors:** Cleonilde Maria do Nascimento, Sheilla Andrade de Oliveira, Otacílio Antunes Santana, Helotonio Carvalho

**Affiliations:** 1 Department of Biophysics and Radiobiology, Biological Sciences Centre, Federal University of Pernambuco, Recife, Pernambuco, Brazil; 2 Department of Immunology, Aggeu Magalhães Institute (IAM), Oswaldo Cruz Foundation (FIOCRUZ), Recife, Pernambuco, Brazil

## Abstract

**Background:**

Lockdowns have been fundamental to decreasing disease transmission during the COVID-19 pandemic even after vaccines were available. We aimed to evaluate and compare changes in air quality during the first year of the pandemic in different cities around the world, investigate how these changes correlate with changes in mobility, and analyse how lockdowns affected air pollutants’ annual means.

**Methods:**

We compared the concentrations of NO_2_, PM_2.5_, and PM_10_ in 42 cities around the world in the first months of the pandemic in 2020 to data from 2016-2019 and correlated them with changes in mobility using Human Development Indexes (HDIs). Cities with the highest decreases in air pollutants during this period were evaluated for the whole year 2020. We calculated the annual means for these cities and compared them to the new World Health Organization (WHO) Air Quality Guidelines. A Student’s t*-*test (95% confidence interval) was used to evaluate significant changes.

**Results:**

Highest decreases in NO_2_, PM_2.5_, and PM_10_ were between -50 and -70%. Cities evaluated for the whole year 2020 generally showed a recovery in air pollution levels after the initial months of the pandemic, except for London. These changes positively correlated with year-long mobility indexes for NO_2_ and PM_2.5_ for some cities. The highest reductions in air pollutants’ annual means were from -20 to -35%. In general, decreases were higher for NO_2_, compared to PM_2.5_ and PM_10_. All analysed cities showed annual means incompliant with the new WHO Air Quality Guidelines for NO_2_ of 10 μg/m^3^, with values 1.7 and 4.3 times higher. For PM_2.5_, all cities showed values 1.3 to 7.6 times higher than the WHO Guidelines of 5 μg/m^3^, except for New Delhi, with a value 18 times higher. For PM_10_, only New York complied with the new guidelines of 15 μg/m^3^ and all the other cities were 1.1 to 4.2 times higher, except for New Delhi, which was 11 times higher.

**Conclusions:**

These data show that even during a pandemic that highly affected mobility and economic activities and decreased air pollution around the world, complying with the new WHO Guidelines will demand a global strategical effort in the way we generate energy, move in and around the cities, and manufacture products.

Covid-19 has imposed serious challenges on health authorities and governments [1]. To minimize disease transmission, countries closed borders, imposed quarantines, and used lockdowns, closing schools, stores, restaurants, and offices. With traffic reduction and many industrial activities halted due to the pandemic, air pollution was supposed to be reduced. At the end of February 2020, satellite data from NASA showed significant drops in NO_2_ levels in China [2]. Later on, NO_2_ level reduction was observed by the European Union Copernicus Programme, from the European Space Agency, in Europe, and India [3,4]. Lower air pollution levels were also reported in several cities like Madrid, Barcelona [5], São Paulo [6], Milan [7], London, Paris [8], New Delhi [9], Santiago [10], and countries or world regions like China [11,12], India [13,14], Western Europe [15], and the USA [16].

Air pollution is responsible for more than 7 million deaths each year [17]. From the several air pollutants, particulate matter (PM) has been the one most widely associated with adverse health effects. The last air quality report from the European Environmental Agency (EEA) indicates that from more than 427 000 deaths attributed to air pollution in Europe in 2019, 72% were linked to PM_2.5_ and 9.4% were linked to NO_2_ [18]. Particulate material, comprising PM_2.5_ and PM_10_ as the major components, are mainly derived from combustion processes, including traffic-related and industrial activities. PM is associated with increased risk of death by ischaemic heart disease, cerebrovascular disease, lung cancer, lower respiratory infections, and chronic obstructive pulmonary disease [19]. NO_2_ is also associated to combustion processes, and calls for more attention about its effects on human health have been issued in countries with large diesel vehicle fleets, like many European countries and India, since these vehicles are the main NO_2_ source in urban environments [20].

Despite other studies describing the effects of lockdowns on air pollution levels, most of them compared either a few cities or many cities in the same country/region of the world. Only a few studies investigated air quality changes over longer periods in 2020, generally focusing on the February-May period. This study aimed to compare changes in NO_2_, PM_2.5_, and PM_10_ among 42 cities on six continents during the first months of the pandemic, compare them with 2016-2019 data, and check how they would correlate with changes in city mobility indexes. We also aimed to study selected cities with higher changes in air pollution for the whole year 2020, compare their NO_2_, PM_2.5_, and PM_10_ annual means with the 2016-2020 period and analyse them according to the new World Health Organization (WHO) air quality guidelines [21].

## METHODS

### Choice of the cities

The 42 cities analysed were chosen based on several criteria: 1) air pollution levels of PM_2.5_ ranging from low to high (**Table 1**); 2) geographical location to represent different regions of the world and/or different regions within a country; 3) COVID-19 epidemiology in order to represent countries which were highly affected and little affected by COVID-19, and thus would be applying different levels of mobility restrictions during the time frame evaluated; 4) availability of historical air pollution data, since whenever possible, official data were used. For inclusion in the list, the existence of data for NO_2_, PM_2.5_, and PM_10_ for previous years (2016-2019) and for 2020 was checked, as well as the consistency of these data.

**Table 1:** Cities analysed on this report, PM_2.5_ annual mean, HDI, and source of air quality data

City	City abbreviations	Country	PM_2.5_ annual mean (μg/m^3^)	HDI	Air quality data source
Adelaide	Ade	Australia	7	0.926	South Australian Government Data Directory: https://data.sa.gov.au/data/dataset
Berlin	Ber	Germany	16	0.932	Senate Department for Environment, traffic and climate protection: https://www.berlin.de/senuvk/umwelt/luftqualitaet/
Londres	Lon	UK	12	0.910	London Air: https://www.londonair.org.uk/
Los Angeles	La	USA	12	0.929	US Environmental Protection Agency: https://www.epa.gov/outdoor-air-quality-data/
Mexico City	Mex	Mexico	22	0.807	Gobierno de la Ciudad de Mexico – Calidad del Aire: http://www.aire.cdmx.gob.mx/default.php
Madrid	Mad	Spain	10	0.925	European Environmental Agency: https://www.eea.europa.eu/themes/air
Milan	Mil	Italy	27	0.847	ARPA Lombardia: https://www.arpalombardia.it/
Moscow	Mos	Russia	14	0.902	Open Data Portal Moscow City Government: https://data.mos.ru/
New Delhi	Del	India	143	0.685	Central Control Room for Air Quality Managemnt – New Delhi NCR: https://app.cpcbccr.com/ccr/#/login
New York	Nyc	USA	7	0.913	US Environmental Protection Agency: https://www.epa.gov/outdoor-air-quality-data/
Paris	Par	France	16	0.930	Air Parif: https://www.airparif.asso.fr/
Rome	Rom	Italy	15	0.812	European Environmental Agency: https://www.eea.europa.eu/themes/air
Buenos Aires	BA	Argentina	NA	0.884	Agencia de Protección Ambiental-Buenos Aires: https://www.buenosaires.gob.ar/agenciaambiental
Santiago	San	Chile	29	0.798	Sistema de Información Nacional de Calidad del Aire: https://sinca.mma.gob.cl/index.php/
São Paulo	Sao	Brazil	17	0.864	Companhia Ambiental do Estado de São Paulo: https://cetesb.sp.gov.br/ar/qualar/
Seoul	Seo	South Korea	26	0.892	Air Korea: https://www.airkorea.or.kr/
Sydney	Syd	Australia	8	0.901	NSW Department of Planning, Industry and Environment: https://www.dpie.nsw.gov.au/air-quality
Venice	Ven	Italy	26	0.913	Agenzia Regionale per la Prevenzione e Protezione Ambientale del Veneto: https://www.arpa.veneto.it/
Addis Ababa	Add	Ethiopia	26	0.683	AirNow – US Department of State: https://www.airnow.gov/international/us-embassies-and-consulates/
Abu Dhabi	Abu	UAE	61	0.793	
Bogotá	Bog	Colômbia	17	0.830	
Dhaka	Dha	Bangladesh	82	0.597	
Dubai	Dub	UAE	54	0.809	
Hanoi	Han	Vietnam	23	0.679	
Jackarta	Jac	Indonesia	45	0.598	
Kampala	Kam	Uganda	104	0.523	
Kathmandu	Kat	Nepal	49	0.524	
Kolkata	Kol	India	74	0.624	
Lima	Lim	Peru	39	0.854	
Manama	Man	Bahrain	60	0.835	
Mumbai	Mum	India	64	0.533	
Ulaanbaatar	Ula	Mongolia	57	0.613	
Chengdu	Che	China	63	0.754	China's air quality online monitoring and analysis platform: https://www.aqistudy.cn/historydata/
Guangzhou	Gua	China	36	0.752	
Hangzhou	Han	China	57	0.746	
Nanjing	Nan	China	48	0.747	
Beijing	Bei	China	73	0.749	
Shanghai	Sha	China	45	0.743	
Shenyang	Shy	China	54	0.741	
Shenzhen	Shz	China	27	0.748	
Tianjin	Tij	China	69	0.738	
Wuhan	Wuh	China	57	0.747	

We analysed PM_2.5_ air pollution data for ten cities in China, including Wuhan, Beijing, and Shanghai; three in India, including New Delhi and Mumbai; cities in other Asian countries, such as Seoul, Jakarta, and Hanoi; cities in Europe such as London, Paris, Milan, and Berlin; cities in the Middle East, such as Dubai and Abu Dhabi; cities in Africa, such as Kampala and Addis Ababa; cities in Australia, such as Sydney and Adelaide; cities in North America, such as Los Angeles, New York, and Mexico City; and South America, such as São Paulo and Lima. PM_10_ and NO_2_ data were analysed for 28 cities around the world.

### Time frame for analysis of air pollution data

Data for NO_2_, PM_2.5_ and PM_10_ were first analysed for the first months of the pandemic, defined as February-April. The choice of this period considered the spread of the pandemic and the consequent lockdowns which happened first in China and then in Europe, USA and all the other regions analysed, according to WHO (21). After these initial months, cities which showed the highest changes in the levels of air pollutants were chosen for a further analysis covering the whole year 2020.

### Air quality and mobility data

Air pollution data were obtained from official environmental agencies, websites that reproduce these data, the EEA, US Environmental Protection Agency or US State Department (US Embassies and Consulates). For Berlin, PM_2.5_ data for 2020 was kindly provided by Dr Paul Herenz (Senate Department for Environment, Transport and Climate Protection). Some of the data for Venice were kindly provided by Patti Salvatore and Luca Zagolin from ARPAV – Dipartimento Regionale Qualità dell'Ambiente-Veneto. NO_2_, PM_10_, and PM_2.5_ data were generally obtained as daily mean values expressed in μg/m^3^. 2016-2019-monthly means were calculated and compared to 2020 to generate % changes. **Table 1** summarizes data source for the analysed cities. PM_2.5_ annual means were obtained from the 2018 WHO Global Ambient Air Quality Database [22], except for Dubai, Manama, and Addis Ababa, which were calculated. Except for US Embassies and Consulates data, air pollution levels represent the mean for at least three to six stationary air quality monitoring stations, depending on the number of stations available. Whenever possible, monitoring stations were chosen according to their locations in different areas across the cities.

Anonymous Google Community Mobility data were obtained from Google Inc. (USA) [23]. Changes in mobility for transit stations and workplaces were chosen since they better represent changes with potential impacts on air pollution. Google Mobility data were not available for Chinese cities and for Addis Ababa.

### Statistical analysis

The Human Development Index (HDI) is composed of indicators of three dimensions of human development: longevity, education, and income. The adjusted Human Development Index (HDI_adj_) adds other variables such as health, education, income, inequality, poverty, employment, environmental sustainability and was adjusted for the city limits in our study. The HDI_adj_ was then corrected by the City Development Index Method [24,25]. For this work, United Nations Development Programme (UNDP) data for 2019 were used for the calculations.

Discriminant analysis was performed to classify cities within groups by HDI_adj_. Variables: HDI_adj_, NO_2_ (from 2016-2019 to 2020), PM_2.5_ (from 2016-2019 to 2020), Workplaces Mobility Changes (% change from baseline), and Transit Stations Mobility Changes (% change from baseline) were used in the analysis. The assumptions of discriminant analysis were observed (multivariate normality, homoscedasticity, multicollinearity, and independence) [26]. Regression analyses were used to examine the relationship between environmental variables and mobility variables (*R^2^*>0.80, *p*<0.05, ɛ<0.05), first, between HDI_adj_ (x) and PM_2.5_ annual mean (y), and second, between PM_2.5_ (from 2016-2019 to 2020) (x) and Workplaces, and Transit stations (y). In the second situation, PM_2.5_ data were normalized by (*x_i_* + *x̅*)2^−1^, according to the DA assumptions [26,27]. Statistical analyses were performed with BioEstat 5.3 package [28]. The data were normalized according to the D´Agostino normality test (*p*>0.01) [29]. The data equalization from sampling methods to metric of data presentation was carried out by the IPCC Metrics & Methodology [30]. The data comparison between the periods (2016-2019 and 2020) was performed using the *t* test (95% Confidence, *p*<0.05). All variables were on a normal distribution.

Regression analysis (*R^2^*) was used to indicate trends between NO_2_ and PM_2.5_ levels and mobility for the same city in a time series through the year 2020. We prioritized linear regression to standardize the trend between the cities (Y ^ = β_0+β_1 · X_i ±ɛ i). In this work, the objective of this analysis was not a perfect fit (>*R^2^* possible), but the trend (*p*<0.05 and ε<0.05) [27].

## RESULTS

### Changes in NO_2_, PM_2.5_, and PM_10_ levels in the February-April 2020

**Figure 1:**
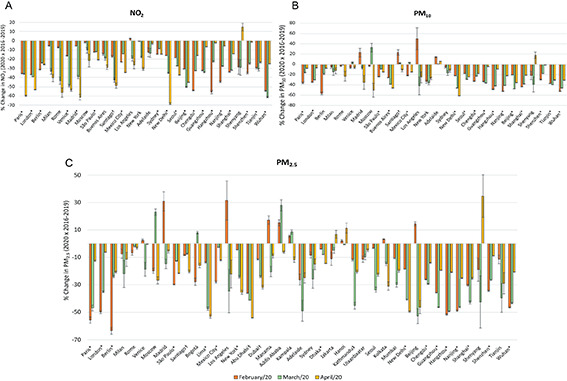
Changes in concentrations of NO_2_ (panel A), PM_10_ (panel B), and PM_2.5 _(panel C) for February-April 2020 compared to 2016-2019. *Cities with *P* < 0.05. †Compared to 2018-2019. ‡Compared to 2017-2019.

We first compared air pollution levels for the first months of the pandemic (February-April) with previous years (2016-2019) to identify the cities most affected by lockdown measures during the first Covid-19 wave. The extent of NO_2_ reductions varied widely among different cities (**Figure 1**, panel A). Most Chinese cities showed the highest reductions in February/March, due to earlier restrictive measures. Most other cities imposed lockdowns in March and higher NO_2_ reductions were observed in April. Among the cities analysed, highest NO_2_ reductions were observed in some Asian and European cities such as Beijing (-50.1%), London (-53.0%), Paris (-59.7%), Wuhan (-61,2%), Madrid (-61.3%), and New Delhi (-68.2%). The results confirmed the statistical difference in NO_2_ levels (*p*<0.05) between the studied periods (2016-2019 and 2020) for 19 out 28 cities with statistically significant changes in NO_2_ (*p*<0.05). No statistically significant NO_2_ changes were found in Moscow, Milan, Rome, Los Angeles, and New York.

New Delhi (-61.4%), Nanjing (-52.9%), and Wuhan (-52.7%) were the cities with highest decreases in PM_10_ (**Figure 1**, panel B). In the case of New Delhi, reductions were progressively higher from February to April. Changes in PM_10_ in Adelaide and Sydney, were not statistically significant. However, both cities showed much higher PM_10_ and PM_2.5_ levels in January compared to 2016-2019 (data not shown) due to extensive wildfires [31]. The results confirmed the statistical difference in PM_10_ levels (*p*<0.05) between the studied periods (2016-2019 and 2020) for 16 out of 28 cities. No statistically significant changes in PM_10_ were observed in Berlin, Rome, Milan, Los Angeles, and New York.

More cities were analysed for PM_2.5_ (**Figure 1**, panel C), since data from US Embassies or Consulates were used for cities with no available data. Paris, London, and Berlin showed highest decreases in PM_2.5_ levels in February (-55.7%, -49.8% and -63.5% respectively). Trends for PM_2.5_ in these cities were similar to those for PM_10_, with significantly lower reduction in April. Among other cities with highest reductions on PM_2.5_ levels were Abu Dhabi (-54%) and New Delhi (-49.6%). Many Chinese cities showed highest decreases on PM_2.5_ levels in February, including Hangzhou and Wuhan (-51.7% and -46.5, respectively) and March, including Beijing and Shanghai (-52.8% and -42.7%, respectively). The results confirmed the statistical difference in PM_2.5_ levels (*p*<0.05) between the studied periods (2016-2019 and 2020) for 18 out of the 41 cities analysed. PM_2.5_ changes were not statistically significant for Rome, Madrid, Los Angeles, Addis Ababa, Jakarta, Kathmandu, and Ulaanbaatar.

### Association between changes in mobility and changes in NO_2_ and PM_2.5_ levels in February-April 2020

**Figure 2:**
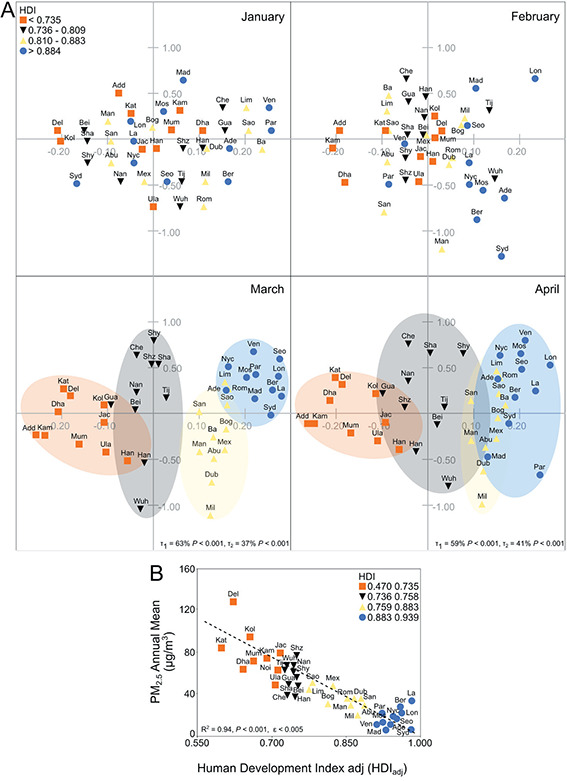
(Panel A) Discriminant analysis for cities according to their HDI_adj_. (Panel B) Association between PM_2.5_ annual means and HDI_adj_.

**Figure 3:**
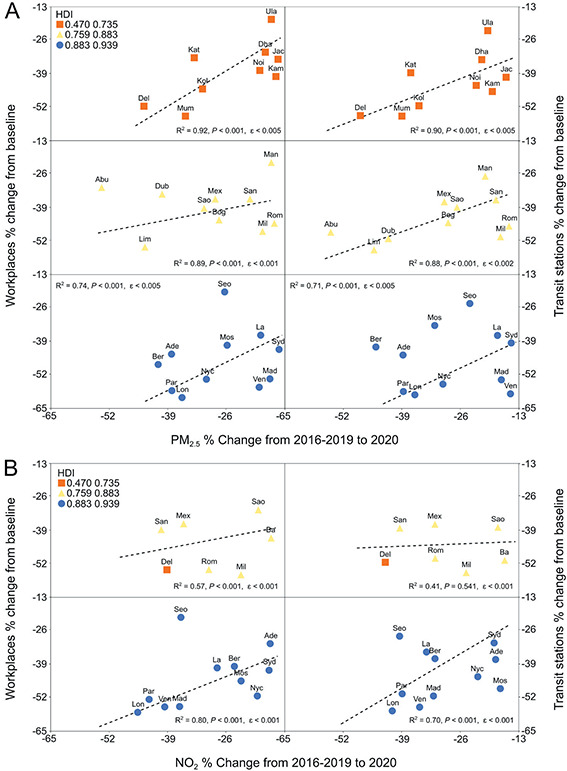
Association between concentrations of PM_2.5_ (panel A) or NO_2_ (panel B) and mobility changes according to cities Human Development Index.

Anonymous GPS data from mobile phones have been used as a convenient indicator for lockdown measures efficiency [32]. With a few exceptions, most cities were already significantly affected by lockdown/social distancing measures by March 2020, showing decreased mobility indexes, (Figure S1, in the Online Supplementary Document). By April 2020, the effect of lockdowns was more evident, with most cities showing -60% to -80% reductions in mobility. Since the decrease in air pollutants was probably associated with lockdowns, we used mobility changes to evaluate a possible association with changes in air pollution among 16 cities, for NO_2_ and PM_10,_ and 28 cities, for PM_2.5_. Discriminant analysis was used to classify cities by HDI_adj_, according to NO_2_ and PM_2.5_ changes, as shown in Figure 2, panel A. In March and April, PM_2.5_ and NO_2_ data grouped into cities with similar HDI_adj_ values, forming four different groups. City development is differentially associated to air pollution since pollutants emission varies in developed and emerging countries [33]. A possible association between PM_2.5_ annual means and HDI_adj_ was also evaluated (**Figure 2**, panel B) showing a clear negative trend between these variables, with highly developed cities showing significantly lower PM_2.5_ annual means. A possible association between Google Mobility Indexes and changes in PM_2.5_ and NO_2_ levels was then investigated. **Figure 3**, panel A shows that reductions in PM_2.5_ levels were clearly associated with changes in Workplace and Transit Stations Mobility according to the city HDI_adj_, with higher R^2^ for cities with lower HDI_adj_. **Figure 3**, panel B shows the same analysis for NO_2_. Only HDI_adj_ strata which showed statistical relevance are show. In this case, cities with higher HDI_adj_ showed the best correlation between decreases in NO_2_ levels and mobility changes.

### Changes in NO_2_, PM_2.5_ and PM_10_ in 2020 for selected cities.

**Figure 4:**
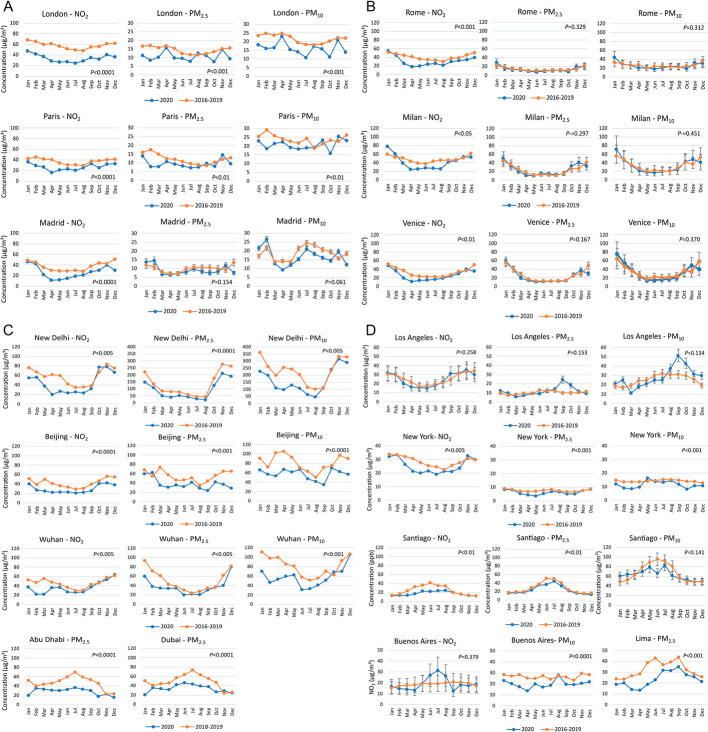
Concentrations of NO_2_, PM_2.5_, and PM_10_ in 2020 and 2016-2019 for cities in Europe (panel A and panel B), Asia and Middle East (panel C), and North and South America (panel D). For Dubai and Abu Dhabi, 2020 data were compared to 2018-2019 means. *P* < 0.05 was considered statistically significant.

The 16 cities with highest decreases in air pollution levels over time in February-April and increasing reductions in this period, were further selected to investigate the whole year 2020 compared to 2016-2019. The levels of air pollutants were lower most of the year for London, Paris and Madrid, with highest decreases generally between February-May (-32% to -61%) (Figure, panel A). However, changes in PM_2.5_ and PM_10_ for Madrid were not statistically significant (*p*>0.05). Among Italian cities, NO_2_ levels were lower during most of 2020. However, changes in PM_2.5_ and PM_10_ were not statistically significant (**Figure 4**, panel B).

All the cities in Asia and Middle East showed decreased levels of all air pollutants throughout most 2020, with highest decreases for NO_2_ and PM_10_ in February-May (-36% to -68%), in the case of New Delhi, Beijing and Wuhan (Figure, panel C). For Abu Dhabi, highest reductions in PM_2.5_ were before the pandemic, in January (-62%) and May-October (-41% to -61%). The pattern of changes in PM_2.5_ levels was similar for Dubai, with highest reductions in January (-60%), and April-October (-26% to -43%).

Among cities in North and South America, overall changes in air pollutants in Los Angeles were not statistically significant (**Figure 4**, panel D). However, massive increases in PM_2.5_ (+82% to +144%) and PM_10_ (+44% to +64%) were observed in September-October due to wildfires [34]. For New York, NO_2_, PM_2.5_ and PM_10_ were lower most of the year, with highest decreases in March-May (-27% to -49%). In the case of Santiago, NO_2_ changes were higher than other pollutants with highest decreases spread over several months (March-August: -29 to -49%), while changes in PM_10_ were not statistically significant. The same was also the case for NO_2_ changes for Buenos Aires, while PM_10_ was decreased most of the year, similarly to what was observed for PM_2.5_ in the case of Lima. Overall, the results confirmed the statistical difference (*p*<0.05) in the data between the studied periods (2016-2019 and 2020) for most of the cities and pollutants analysed.

### Association between changes in NO_2_ or PM_2.5_ and changes in mobility indexes in selected cities in 2020.

**Figure 5:**
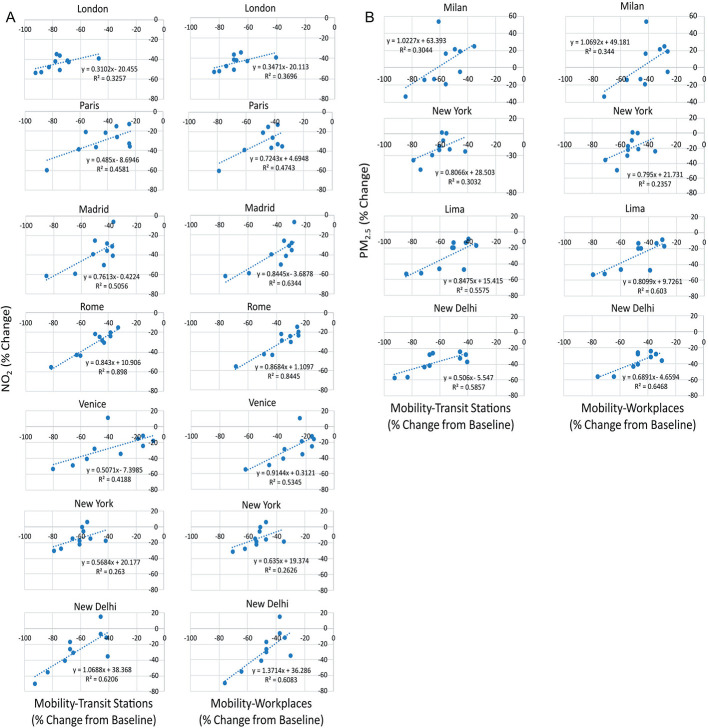
Association between concentrations of NO_2_ (panel A) or PM_2.5_ (panel B) and mobility changes in March-December 2020. *P* < 0.01; ɛ<0.05.

Google mobility data were further analysed for the whole year 2020 (Figure S2 in the **Online Supplementary Document**). Transit station and workplace changes were still consistently lower up to December for some cities, including Abu Dhabi, London, Milan, and New York. In other cities, such as Dubai, Santiago, Madrid, and New Delhi, transit stations changes were progressively lower until December. A possible association between changes in mobility and changes in air pollution over time was then investigated. Chinese cities were not included due to the lack of mobility data. Many of the cities showed an association between transit stations or workplaces mobility and NO_2_ changes (**Figure 5**, panel A), with the strongest associations for Rome (*R^2^* = 0.89 for transit stations, *R^2^* = 0.84 for workplaces), New Delhi (*R^2^* = 0.62 and *R^2^* = 0.61), Madrid (*R^2^* = 0.51 and *R^2^* = 0.63), Paris (*R^2^* = 0.46 and 0.47), and Venice (*R^2^* = 0.42 and *R^2^* = 0.53). London and especially New York showed a weaker association between mobility and NO_2_ changes. Milan, Los Angeles, Santiago, and Buenos Aires did not show any association between changes in mobility and NO_2_. An association between mobility indexes and PM_2.5_ levels was observed only for a few cities (**Figure 5**, panel B), with strongest associations for New Delhi (*R^2^* = 0.59, transit stations and *R^2^* = 0.65, workplaces) and Lima (*R^2^* = 0.56 and *R^2^* = 0.60), and weaker associations for Milan. There was no apparent association between changes in mobility and PM_2.5_ for Dubai, Abu Dhabi, Paris, London, Rome, Venice, Los Angeles, Madrid, Santiago, and Buenos Aires.

### Effect of Covid-19 lockdowns in PM_2.5_, PM_10_ and NO_2_ annual means

**Figure 6:**
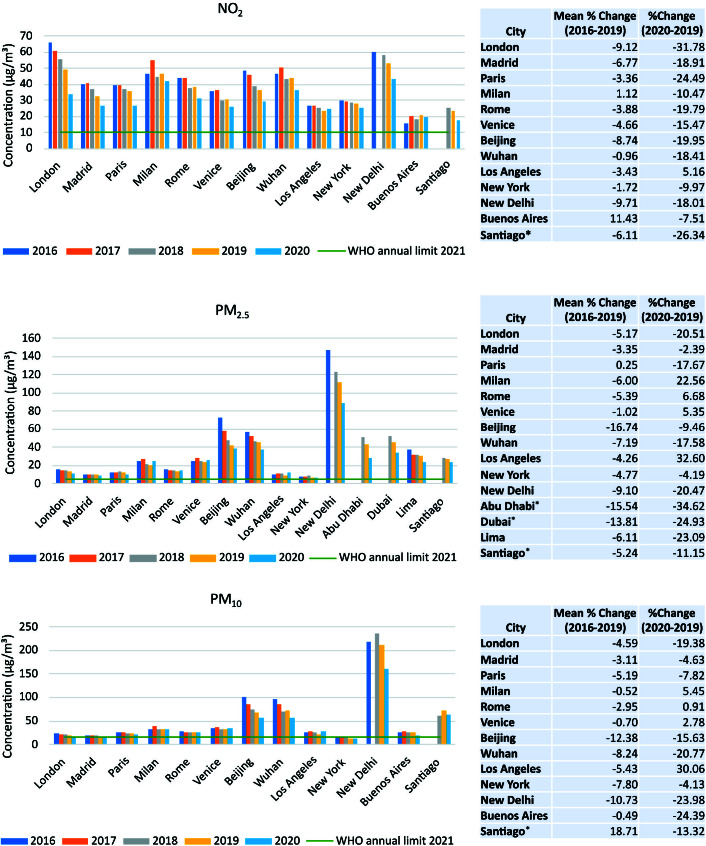
Annual mean concentrations for NO_2_, PM_2.5_, and PM_10_ during 2016-2020 and comparison with the new WHO Air Quality Guidelines. Inner tables show the mean percent change in annual means from year to year for 2016-2019 and for 2020-2019. *Compared to 2018-2019. For New Delhi, data from 2017 were not available for the whole year and were not included in the analysis.

We also investigated how differences in air pollution levels in 2020 affected annual PM_2.5_, PM_10_ and NO_2_ concentrations, compared to 2016-2019. **Figure 6** shows many cities have progressively reduced NO_2_ levels between 2016 and 2020, which is probably related to efforts to decrease air pollution, especially where NO_2_ levels have been historically higher. To compare year-to-year variation, percent changes between subsequent years were calculated and the mean change from 2016-2019 was compared to 2020 (**Figure 6**). Decreases in NO_2_ annual means were much higher in 2020, especially for London, Paris, and Santiago (-24% to -32%) while most cities showed 2016-2019 decreases in annual means lower than -10%. Los Angeles was the only city with higher NO_2_ levels in 2020, despite the small difference (+5%). As shown for NO_2_, most cities showed progressive decreases in PM_2.5_ annual means in 2016-2019, and higher decreases in 2020. Highest decreases (-18% and -35%) were observed in London, Paris, Wuhan, New Delhi, Abu Dhabi, Dubai, and Lima. However, Rome, Milan, and Venice showed no decrease in PM_2.5_ annual mean, instead, there was an increase as high as +23% in the case of Milan. An increased PM_2.5_ annual mean in 2020 was also observed in Los Angeles due to wildfires. In the case of PM_10_, there was also a general decrease along the years, which was particularly higher in 2020 in the case of London, Wuhan, New Delhi, and Buenos Aires (-19% to -24%). Again, Rome, Milan, and Venice showed increases in PM_10_ annual mean, but smaller than the increases in PM_2.5_. PM_10_ annual mean for Los Angeles was +30% higher in 2020 compared to 2019, in agreement with increases in PM_2.5_.

## DISCUSSION

Despite its serious impacts on the world, the COVID-19 pandemic had a positive effect on air pollution. Maximum decreases in air pollution levels described here were, in general, similar to other reports. For NO_2_, a -58.4% decrease was described for Hangzhou [35], -62.0% for Madrid [5], -53,6% for London, -39.0% for Milan [8], and -60% for Wuhan [36]. For some cities, other reports have found different values for changes in air pollution, what may be related to differences on the choice of monitoring stations, the specific time frame analyzed and/or used for comparison. Thus, a -40% decrease was described for Beijing (-40%), compared to 2018 [36], -51.5% for Paris, compared to 2017-2019 values [8], and -61% for New Delhi, compared to 2017-2019 [14].

Interestingly, even though some cities were studied in other reports, this is the first one to describe changes in air pollution levels due to Covid-19 restrictions in Venice. This is surprising, since NO_2_ annual mean in Venice is similar to Paris, while PM_2.5_ and PM_10_ means are much higher. Besides this, Venice is one of the cities with worst air qualities among more than 300 cities in Europe, based on PM_2.5_ levels [37], which may be associated to its large dependency on diesel-powered water transport (Vaporetto) and to cruise ships which reach the Venice Lagoon.

For PM_10_, similar maximum reductions were described for New Delhi (-57%) [14], London (-34%) [38] and Wuhan (-55%) [39]. For PM_2.5_, highest decreases close to the reported here were observed for Paris (-58%), and London (-56%) [39], New Delhi (-53%) [14], Shanghai (-41%) [40] and Wuhan (-38%) [36]. Despite the absence of statistically significance, the smaller decreases/increases observed for some cities were also described by others for Addis Ababa, Dhaka, Kampala, Jakarta, and Ulaanbaatar [41]. Other authors analysed the effects of COVID-19 restrictive measures in Sydney and Melbourne, but as far as we know, this is the first study to analyse data for Adelaide.

Beijing was the city with highest discrepancies in PM_2.5_ changes compared to other reports. However, the way data were compared was different among these studies, using comparison against 2015-2019 [39], 2018-2019 [41], 2017-2019 [42] and 2016-2019 for the present report.

Many cities in low- and middle-income countries have poor urban planning, disorganized traffic, lack regular vehicular emission inspection and have older cars compared to high income countries. However, many cities in high-income countries stimulate dispersion of households away from the city centres, emphasize more environmentally friendly and efficient modes of transportation, and restrict the access to city centres creating low and ultralow emission zones [43]. These aspects make the association between air pollutants and mobility vary among cities in low- and middle- compared to high income countries [44]. We used HDI_adj_ to group cities and compensate for such variables, allowing for a better analysis of associations between mobility and air pollution. Sokhi et al. [39] found a positive correlation between mobility data and NO_2_ concentrations (*R^2^* = 0.513) but did not find a correlation for PM_2.5_ and argued that atmospheric chemical reactions leading to the formation of secondary aerosols might explain this. Venter et al. [45], only found a correlation between workplaces mobility data and NO_2_ and attributed this to the fact that, in many countries, PM_2.5_ is more associated with residential and regional energy generation and agriculture. Despite the reasons addressed by these authors, the approach used here was successful in also confirming an association between changes in mobility and PM_2.5_ levels. Besides this, the *R^2^* values for both pollutants were higher for most HDI_adj_ strata analysed here. Interestingly, when cities were analysed throughout 2020 and the regression analysis was performed between same city data, more cities showed a relevant association for NO_2_ than for PM_2.5_. This is probably linked to differences in the main sources of PM_2.5_ and NO_2_ in different cities.

Data analysis for the whole year 2020 shows that, even though many of these cities extended or reapplied lockdowns after this period, as confirmed by the analysis of mobility indexes, the major effect of these measures on air pollution levels was observed on the first months of the pandemic, February-May. An interesting exception is London, where NO_2_ levels were much lower, during the whole year. Other cities, like Paris and Madrid, also showed lower NO_2_ levels during the whole year but decreases were much smaller for some of the months. Highest reductions in NO_2_ were observed for some European cities such as London, Paris, and Madrid, as well as for New Delhi. The general trend for PM_2.5_ levels at the beginning of the pandemic was quite different compared to NO_2_. Cities like Paris, London, and Berlin showed decreasing reductions in PM_2.5_ levels from February to April while only a few cities like Lima, Abu Dhabi, Dubai, and New Delhi showed increasing reductions over these months. The major effects of lockdowns on PM_2.5_ levels were visible between March and April. London, Beijing, New Delhi, Abu Dhabi, and Dubai, however, showed decreased PM_2.5_ levels throughout most of the year. In general, decreases in PM_2.5_ levels were smaller when compared to NO_2_ which was especially the case for Italian cities. As expected, trends in PM_10_ were similar to PM_2.5_, as observed for Paris, London and Berlin, for example, on the first months of the pandemic and for most cities analysed for the whole year 2020.

Successive decreases in NO_2_ annual means, and higher decreases in 2020, have driven many of the cities below or close to WHO guidelines (2005) of 40 μg/m^3^. Milan and New Delhi were the only cities slightly above these guidelines. However, compared to the new stricter WHO guidelines (2021) [46], all the cities analysed showed 2020 NO_2_ levels 1.7-4.3 times higher than the 10 μg/m^3^ guideline. Even after successive decreases in PM_2.5_ annual means and higher decreases observed in 2020, many of the cities still did not comply with the WHO guidelines (2005) of 10 μg/m^3^. With the new WHO guidelines (2021) of 5 μg/m^3^, Milan and Venice showed five times higher PM_2.5_ levels in 2020, Abu Dhabi, Dubai, Lima, and Santiago 4.7-6.8 times higher, Beijing and Wuhan 7.5 times higher, and New Delhi showed levels 18 times higher than the new WHO guidelines. Madrid, Paris, and New York were the only cities with PM_2.5_ annual means lower than WHO guidelines (2005) but were above the new guidelines (2021), with 2020 pm_2.5_ levels 1.3-1.9 times higher. In 2020, only London, Madrid, New York, and Buenos Aires complied with the older WHO guidelines (2005) for PM_10_ of 20 μg/m^3^. With the new guidelines (2021) of 15 μg/m^3^, this happened only for New York, while London, Madrid and Buenos Aires were barely above it. Milan, Rome, Venice, and Los Angeles were 1.7-2.2 times higher; Beijing, Wuhan, and Santiago, 3.8-4.2 times higher; and New Delhi, 11 times higher than WHO guidelines. Despite using different data sets, timeframes, and analysing different cities, Carvalho [47] showed that even cities with lower air pollution levels will require additional efforts to satisfy the new WHO Air Quality Guidelines. However, the challenge will be much harder for cities with higher air pollution levels, with many of them in need for at least 90% reduction in PM_2.5_ levels, compared to 2018 WHO data, including Beijing, Ulaanbaatar, and New Delhi.

It is important to mention that this study has some limitations. Changes in meteorological variables may impact the concentration of air pollutants and a specific compensation for local meteorological factors was not carried out. Instead, we used year to year comparison to account for changes in meteorological variables. Different authors have also reported the impacts of lockdowns on air pollution using a year-to-year comparison as we have done here [36,41], to account for these changes and our results are in line with these as well as other studies which considered specific local meteorological factors [38-40]. Another limitation of the study is the fact that Google Mobility data only accounts for Google users, while a fraction of the population was not considered. Besides this, the use of Google Mobility was not possible for Chinese cities, due to lack of data availability, but these data were not available for other mobility indexes as well, such as Apple Mobility.

The results reported here corroborate these conclusions and show that even with the COVID-19 pandemic, the decrease in air pollutant levels was limited if we consider the whole year 2020, since most cities showed a “recovery” in air pollution levels after the most restrictive months of the pandemic.

## CONCLUSIONS

Lockdowns have been a valuable containment strategy for COVID-19, used even after vaccines were available, to maintain a low numbers of new cases, hospitalizations, and deaths. For many of the cities analysed, 2020 annual means for NO_2_, PM_2.5_ and PM_10_ were much smaller than for 2016-2019, indicating that altogether, even when restrictions were suspended, the increase in the levels of these pollutants was not enough to counteract the decrease previously observed. However, even with the reductions in annual means, many cities were far for complying with the new WHO Air Quality Guidelines, especially for NO_2_.

## Additional material:


Online Supplementary Document
