# Supplementary material for: Changes in air pollution due to COVID-19 lockdowns in 2020: Limited effect on NO2, PM2.5, and PM10 annual means compared to the new WHO Air Quality Guidelines
Source: J Glob Health. 2022 Nov 21;12:05043. doi: 10.7189/jogh.12.05043 (PMC9677514; doi:10.7189/jogh.12.05043)

Figure S1. Changes in mobility indexes for transit stations and workplaces in March (A) and April (B) 2020.

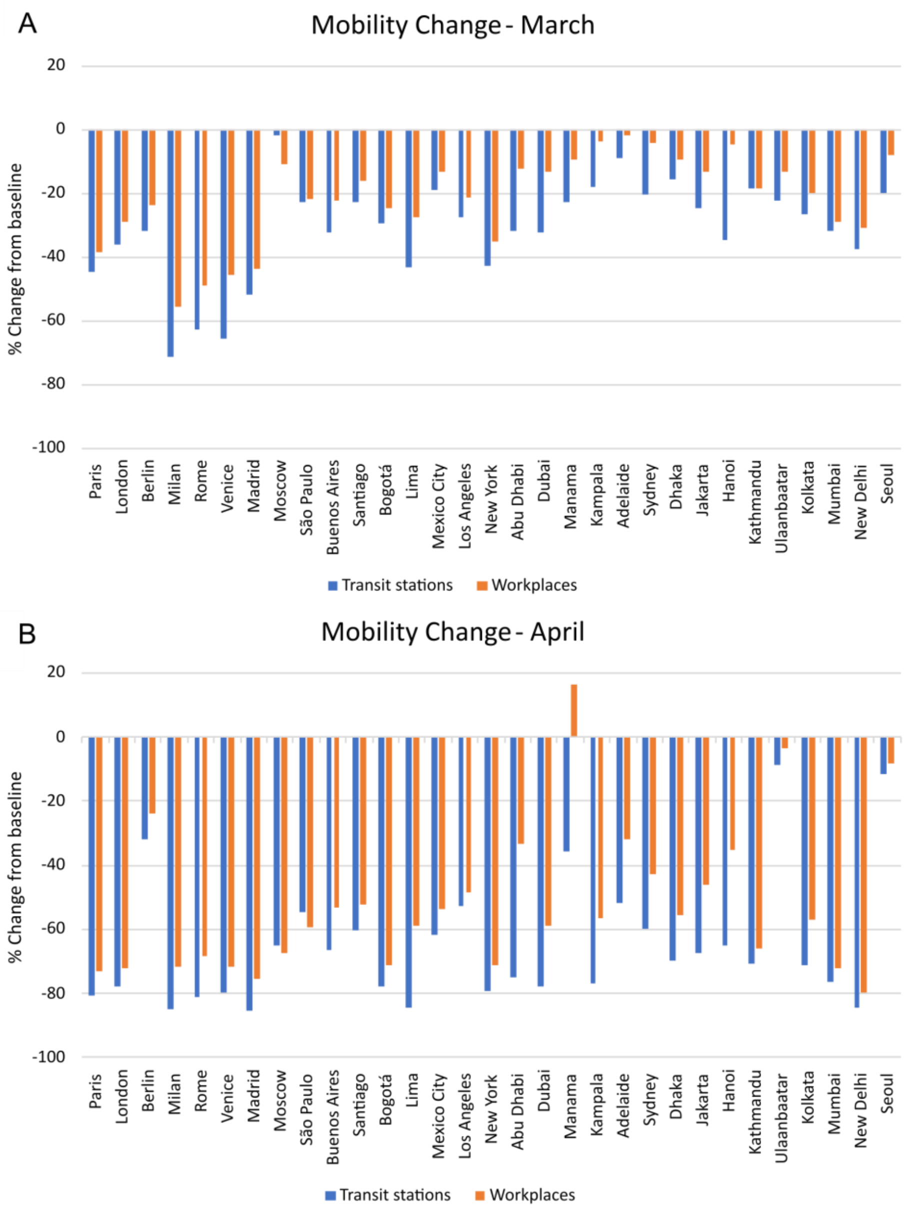

Figure S2. Changes in mobility indexes for transit stations and workplaces from March to December 2020.

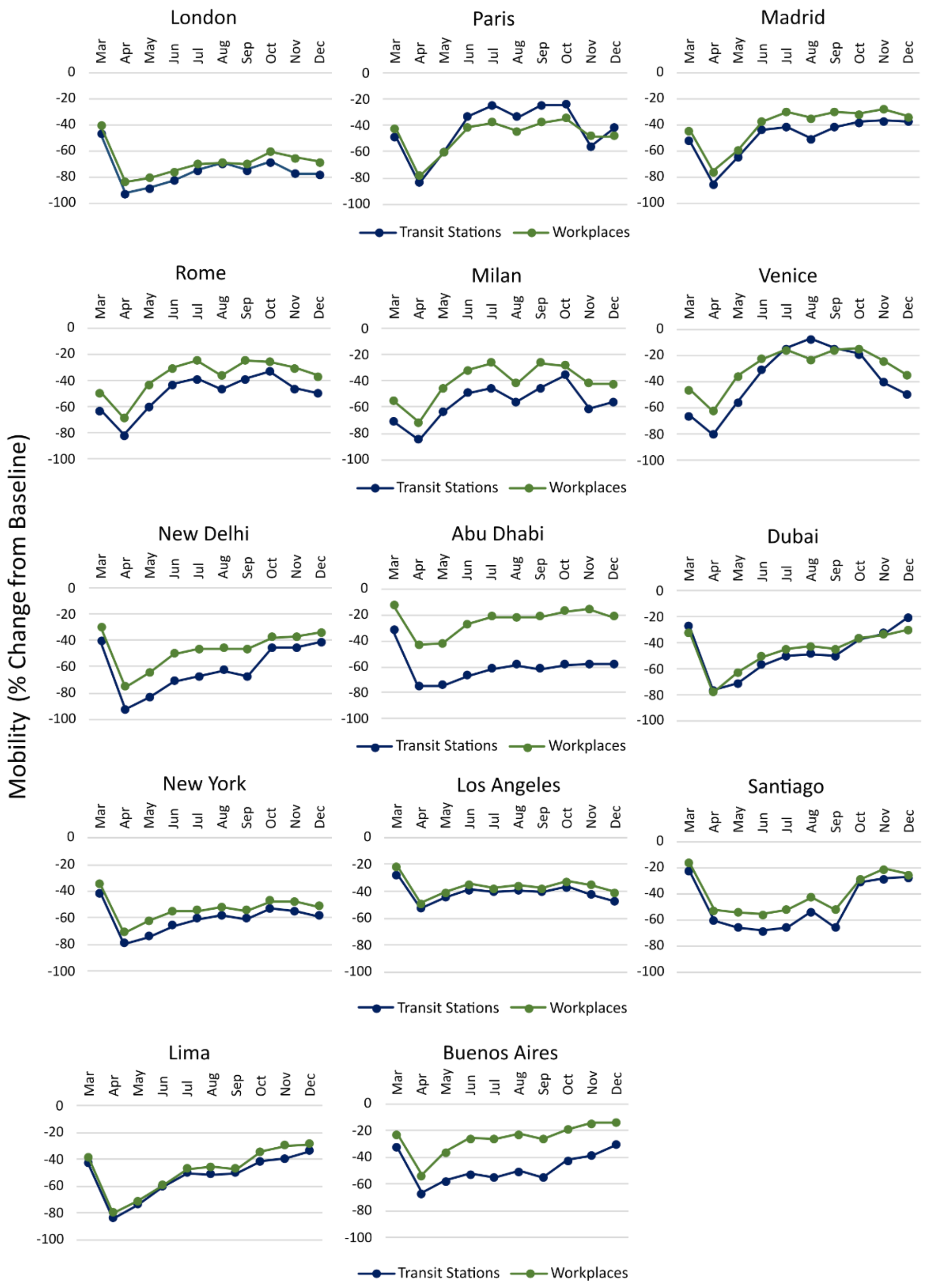

Supplement: Online Supplementary Document [file jogh-12-05043-s001.pdf]
